# Thermal and optical properties of P3HT:PC70BM:ZnO nanoparticles composite films

**DOI:** 10.1038/s41598-023-47134-4

**Published:** 2024-01-02

**Authors:** B. Hajduk, P. Jarka, H. Bednarski, M. Godzierz, T. Tański, M. Staszuk, P. Nitschke, B. Jarząbek, M. Fijalkowski, K. Mazik

**Affiliations:** 1 Centre of Polymer and Carbon Materials, Polish Academy of Sciences, 34 Marie Curie-Skłodowska Str., 41-819 Zabrze, Poland; 2 Department of Engineering Materials and Biomaterials, Silesian University of Technology, 18a Konarskiego Str., 41-100 Gliwice, Poland; 3 Institute for Nanomaterials, Advanced Technologies and Innovation, Technical University of Liberec, Studentská 1402/2, 461 17 Liberec, Czech Republic

**Keywords:** Polymers, Nonlinear optics, Nanoparticles, Engineering, Materials science, Physics

## Abstract

The results of studies on the influence of zinc oxide nanoparticles (ZnO-NPs) on the structural, thermal and optical properties of thin films of mixtures of phenyl-C71-butyric acid methyl ester (PCBM) with poly[3-hexylthiophene] (P3HT) of various molecular weights are described in this article. The structural properties of the layers of: polymers, mixtures of polymers with fullerenes and their composites with ZnO-NPs were investigated using X-ray diffraction. Whereas their glass transition temperature and optical parameters have been determined by temperature-dependent spectroscopic ellipsometry. The presence of ZnO-NPs was also visible in the images of the surface of the composite layers obtained using scanning electron microscopy. These blends and composite films have also been used as the active layer in bulk heterojunction photovoltaic structures. The molecular weight of P3HT (Mw = 65.2; 54.2 and 34.1 kDa) and the addition of nanoparticles affected the power conversion efficiency (PCE) of the obtained solar cells. The determined PCE was the highest for the device prepared from the blend of P3HT:PCBM with the polymer of the lowest molecular weight. However, solar cells with ZnO-NPs present in their active layer had lower efficiency, although the open-circuit voltage and fill factor of almost all devices had the same values whether they contained ZnO-NPs or not. It is worth noting that thermal studies carried out using temperature-dependent ellipsometry showed a significant effect of the presence of ZnO-NPs on the value of the glass transition temperature, which was higher for composite films than for films made of a polymer-fullerene blend alone.

## Introduction

Photovoltaic devices based on organic electronics are becoming a viable alternative to silicon solar cells^1^. However, their weakness so far is lower efficiency and thermal stability. Intensive research is being conducted to overcome these difficulties^1–6^. Particular attention was paid to polymeric devices based on materials processed in the liquid phase. This allows the production of photovoltaic cells with simple printing techniques, while covering quite large surfaces, including flexible substrates^5^, and maintaining low production costs. The widespread interest in organic photovoltaic (OPV) devices causes continuous development of this technology, and thus—a gradual increase in their efficiency. While in 2009 the average efficiency of cells was c.a. 6%, now it is over 16%^4^ and even 18%^5^. As a result, several areas of research on optimizing the parameters of organic solar cells (OSCs) have emerged, which are widely discussed in review and scientific articles^1–14^. One of the intensively developed research directions in recent years is the study of the influence of the morphology of the active layer on the operation of OPV devices^13,14^. This line of research includes not only works related to the study of the impact of changes in key parameters, such as the impact of solvents, molecular weight of polymers or physical conditions of layer deposition. But this research trend also includes efforts related to the introduction of various inorganic interlayers or even nanoparticles directly into the active layer and the analysis of their impact on the efficiency of photovoltaic cells. This, for instance, includes research involving the use of inorganic semiconductors such as TiO_2_ and/or ZnO as interlayers to improve electron transport in OSCs^15–19^. On the other hand, metal oxide nanoparticles can be added directly to P3HT:PCBM blends. For example, as in the work of Ikram et al.^20^, where a mixture of ZnO and TiO_2_ nanoparticles was added to the P3HT:PC60BM mixture. The samples in these studies were prepared with an increasing content of nanoparticles, balanced by a decrease in PCBM. The highest *PCE* was 3.1% with an optimal nanoparticle content of 17.5%. While in the work of Chonsuty et al.^21^, only ZnO-NPs was added to the P3HT:PCBM mixture. In this case, the *PCE* of the obtained solar cells was the highest for the cell in which the concentration of ZnO-NPs was 5 mg/ml. In the context of the research discussed here, it is worth mentioning that fullerene-based photovoltaic cells are commonly used as bulk hetero-junction (BHJ) comparison devices^22,23^ to test other devices, e.g. made of various organic materials. In the construction of these devices, P3HT is most often used as a donor, and PC61BM or PC71BM as acceptors; these polymer: fullrene structures are extensively described in the literature^24–26^. The efficiency of this type of OSCs is usually in the range of several percent. Another important factor is the thermal stability of the OSC, which directly affects their service life. This fact gives a strong impulse to conduct research on the thermal stability of materials used in OSCs, e.g.^6,23–32^, and on material limitations and the possibilities of their improvement^6^. As can be seen from the discussion above, the thermal properties of the materials used as the active layer in OPV devices have a significant impact on their thermal stability and service life. However, to the best of our knowledge, much less is known about how the addition of nanoparticles to the OSCs active layer affects their thermal properties. Therefore, in this article we will focus on studies that shed more light on this topic. To this end, we conducted detailed studies of the structural and optical properties of P3HT:PC70BM mixed layers and their composites with ZnO-NPs in order to characterize them thoroughly. We also prepared the corresponding OSCs, i.e. with and without ZnO-NPs in the active layer, to test and compare their performance. All this gives a solid basis for research using spectroscopic ellipsometry with variable temperature. Especially for studying the influence of ZnO-NPs on the value of the glass transition temperature (*T*_*g*_) of these layers. This is possible because the *T*_*g*_ of thin layers of organic semiconductors can be determined directly from the temperature dependence of ellipsometric angles *Ψ* or *Δ*^8–10^ or changes in physical quantities, such as their thickness or refractive index^27–32^ caused by temperature changes. Additionally, considering that the *T*_*g*_ of polymer films may depend on the molecular weight of the polymer, which is due to the Flory-Fox relationship, we performed these studies for three P3HTs with different molecular weights.

## Materials and methods

**Figure 1:**
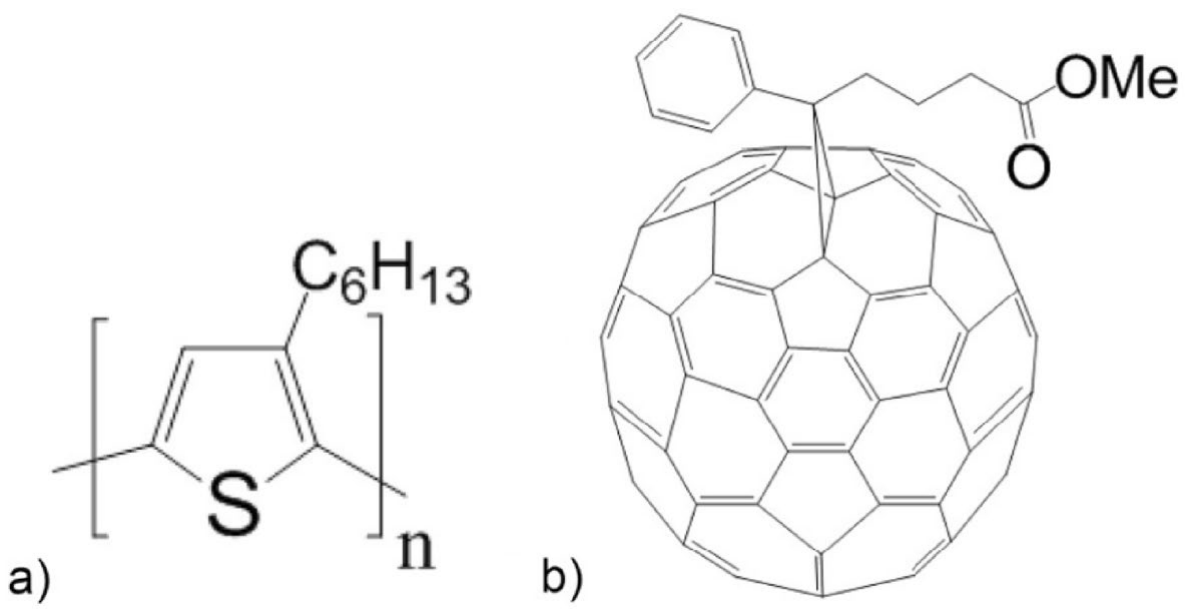
Chemical structures of (**a**) P3HT and (**b**) PC70BM.

The materials here used are: poly(3-hexylthiophene-2,5-diyl) P3HT M102—**P3HT1** with molecular weight Mw = 65.2 kDa (95.7 RR), poly(3-hexylthiophene-2,5-diyl) P3HT M103—**P3HT2** with molecular weight Mw = 54.2 kDa (94.2 RR), poly(3-hexylthiophene-2,5-diyl) P3HT M106—**P3HT3** with molecular weight Mw = 34.1 kDa (94.7 RR), [6,6]-Phenyl-C71-butyric acid methyl ester—M114—**PC70BM** with molecular weight Mw = 1031 g/mol (above 99 wt% purity) and **ZnO-NPs** powder. **PEDOT:PSS**—HTl Solar. All these materials have been supplied by Ossila. The chemical structures of used organic compounds are shown in Fig. 1.

Samples from chloroform solutions with a constant weight concentration of 20 mg/ml were prepared. The content of P3HT to PC70BM was 1:1 in all the blends and composite films. In solutions, where the ZnO-NPs has been added, the nanoparticles weight concentration was 10%. All the solutions were homogenized at 16 kJ for 10 min, using a Bandelin Sonoplus homogenizer. Next, thin films were deposited on silicon wafers covered with 400 nm SiO_2_ film and on ITO substrates, using the spin-coating method with 2000 rpm spinning rate, during time t = 60 s. The organic solar cells structures were prepared in step, by step method. The ITO substrates were rinsed in isopropyl alcohol at first. Next, the PEDOT:PSS layer was deposited with spin-coating method. The technical parameters of spin coating were the spinning rate (V_S_ = 5000 rpm) and the spinning time (t = 60 s). The PEDOT:PSS layer was annealed at 120 °C, during 20 min, to evaporate remaining water. After that, the active layer of blend or blend/composite was deposited, using spin-coating as well.

The ellipsometric measurements were carried out using the SENTECH SE850E spectroscopic ellipsometer (SENTECH Instruments GmbH, Berlin, Germany), operating on the Spectra Ray 3 software and working within the 240–2500 nm wavelength, λ, range. Additionally, the variable-temperature spectroscopic ellipsometry (VTSE) measurements have been performed using variable temperature chamber, operating at lowered pressure, and an INSTEC mK1000 temperature controller (Instec, Inc., Boulder, CO, USA). The control of temperature is precisely maintained by the controller using an electric heater and liquid nitrogen circuit. For temperature-dependent ellipsometric measurements the following protocol has been used. Every sample was annealed at 300 °C for 2 min and then was cooled to − 50 °C under vacuum 10^–1^ Tr. The temperature-dependent measurements were performed in 240–930 nm wavelength range, with 10 s time intervals between single measurements. All the measurements were performed under vacuum 10^–1^ Tr and using the heating rate of 2 °C/min.

All the optical results of as-prepared samples were performed using variable-angle spectroscopic ellipsometry VASE), in 240–2500 nm range. The angle of incidence of the light beam was in the range of 40–70°, and the measurement was performed with the step of 2.5°.

X-Ray diffraction studies were performed using the D8 Advance diffractometer (Bruker, Karlsruhe, Germany) with Cu-Kα cathode (λ = 1.54 Å). Due to relatively high film thickness of sample (around 1000 nm), the classic Bragg–Brentano geometry measurement was applied. The scan rate was 1.2°/min with scanning step 0.02° in range of 2–60° 2Θ (dwell time 1 s). Background subtraction, occurring from air scattering, was performed using DIFFRAC.EVA program.

SEM images were obtained using the Zeiss Supra 35 scanning electron microscope, which accelerating voltage is in 2–4 kV range, work distance in 3–3.5 range. The images were collected using In-Lens mode (for flat and nanometric samples).

The parameters of obtained photovoltaic solar cells were measured using dedicated testing board and solar simulator SS80AAA (Ossila).

## Results and discussion

### X-Ray diffraction

**Figure 2:**
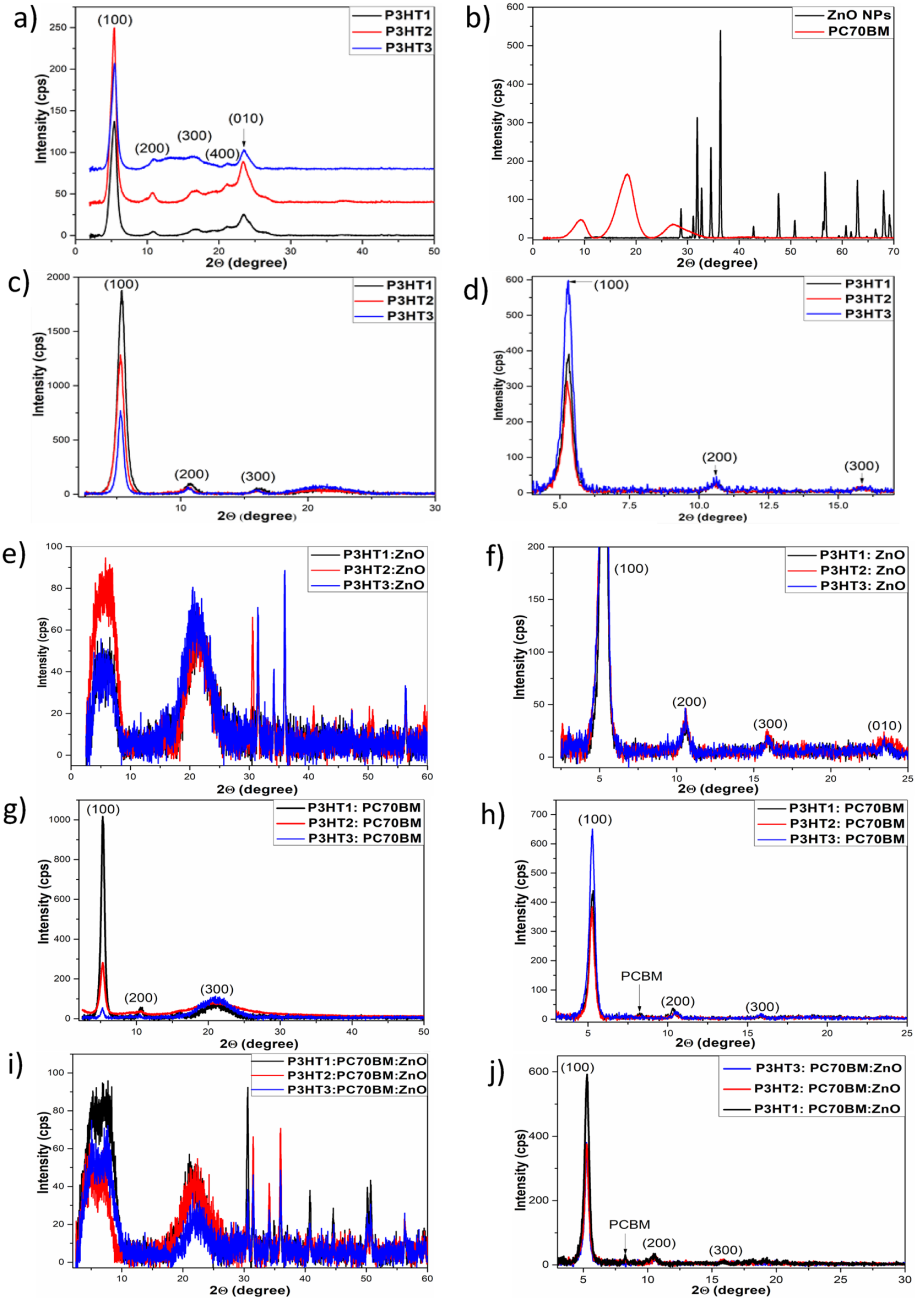
XRD patterns of: P3HT1-3 (**a**), PC70BM and ZnO-NPs (**b**)powders, P3HTt1-3 films before (**c**) and after annealing (**d**), P3HT1-3:ZnO films before (**e**) and after annealing (**f**), P3HT1-3:PC70BM films before (**g**) and after annealing (**h**), P3HT1-3:PC70BM:ZnO films before (**i**) and after annealing (**j**).

In order to examine the presence of ZnO-NPs in composite films, impact of thermal annealing and to determine the effect of the polymer molecular weight on the microstructure of composite films, we conducted detailed X-ray diffraction studies, which included powders of purchased materials and films obtained from their mixtures with an increasing number of individual components. Namely: ZnO nanoparticle powder and one-component P3HTi (*i* = 1–3) and PC70BM powders and films; two-component films P3HTi + PC70BM70 and P3HTi + ZnO-NPs; and ternary composite films P3HTi + PC70BM70 + ZnO-NPs, where P3HTi is P3HT with different molecular weights; in particular: Mw = 65.2, 54.2 and 34.1 kDa for P3HT1, P3HT2 and P3HT3, respectively. The powders of the materials were tested to facilitate the interpretation of more complex results obtained for films of their mixtures. The corresponding XRD spectra are shown in Fig. 2a–b. Looking at the spectra of the P3HTi powders, it can be seen that they are largely crystalline with a dominant peak at 2θ = 5.25˚ and less intense peaks at 10.5, 15.75, 22 and 24°. Moreover, it can be seen that there is a clear correlation in the positions of the first three peaks, namely that the 2θ value of second and third peaks are correlated with the position of the first peak. Since we are using the Bragg–Brentano diffraction geometry the presence of the crystallographic planes parallel to the sample surface is examined. Bragg's law relates the positions of the peaks, here given by 2θ, to the distance between the respective diffraction planes of the crystal. It can therefore be used to confirm that these peaks are from diffraction on a common family of crystallographic planes. In our case, the strongest peak at 2θ = 5.25° corresponds to a distance of 16.8 Å, indicating the presence of an ordered edge-on arrangement of P3HT chains^5,7^. In turn, the position of the other two peaks, corresponding to the distance between the planes c.a. 3.5 A indicate on the π stacking of conjugated P3HT units. Note also that small differences in the degree of crystallinity depending on the molecular weight of the polymer are already visible here. In turn, the ZnO nanopowder is highly crystalline with clearly visible peaks coming from reflections on closely located crystallographic planes, in the 2θ range from 27 to 37°. On the contrary, PC70BM70 powder is much more amorphous, its XRD scan shows three broad contours, the largest of which is the middle one extending from 12 to 22° in the 2θ angle range. The crystallite sizes of P3HT1, P3HT2 and P3HT3, ZnO-NPs and PC70BM powders calculated by Rietveld refining are shown in Table 1.

**Table 1:** Lattice parameters, crystallite size and lattice strain calculated for examined Nb_2_O_5_, ZnO, TiO_2_ and M102, M103 and M106 P3HT powders and PC70BM C60 and C70 powders using Rietveld refinement.

Powder	Space group	Lattice parameters, ICDD (Å)	Lattice parameters, calculated (Å)	Crystallite size (nm)	Lattice strain (%)
ZnO (00-036-1451)	P6_3_mc	a = 3.2498	a = 3.250	79 ± 1	0.06 ± 0.01
		c = 5.2066	c = 5.207		
P3HT1	Orthorhombic, with most likely Pba2 symmetry		a = 10.38	6 ± 1	–
			b = 6.33		
			c = 16.58		
P3HT2		a ≈ 10.3	a = 10.43	8 ± 1	–
		b ≈ 6.6	b = 6.36		
		c ≈ 16.3	c = 16.63		
P3HT3			a = 10.38	9 ± 1	–
			b = 6.39		
			c 16.76		
PC70BM C60	P2_1_/n		a = 13.42	95 ± 10	–
			b = 15.15		
		a = 13.47	c = 19.05		
		b = 15.14	β = 107.01°		
PC70BM C70		c = 19.01	a = 13.55	7 ± 1	–
		β = 106.9°	b = 15.19		
			c = 18.89		
			β = 107.39°		

Moving on to the description of results for films, let us note that the annealing of organic films after their deposition is routine for solvent removal. Therefore, for greater clarity, the results of the XRD spectra for the films are presented in pairs, i.e. for films before and after thermal treatment at *T* = 120 °C. Results for neat polymer films are shown in Fig. 2c–d. In non-annealed films, Fig. 2c, there are visible systematic differences in the intensity of the main peak and the presence of broad contours, extending from 2θ = 16 to 26°, characteristic of the amorphous region, here corresponding to the inter-planar distances characteristic for the π stacking. The polymer film with the lowest molecular weight, i.e. P3HT3, is by far the most amorphous. It has an amorphous contours with the largest area and a main peak with the lowest area. In turn, Fig. 2d, shows that the annealing had the greatest effect on the P3HT film, with the lowest molecular weight, so that after this process it shows the best order. Attention should also be paid to the complete disappearance of the amorphous area in the annealed films. This was due to the improvement of π stacking, most likely in the direction perpendicular to the lamellas stacking.

Currently, it is interesting to look at the effect of the ZnO-NPs addition on the microstructure of the polymer composite film. The corresponding XRD spectra of the P3HTi:ZnONPs composite films are shown in Figs. 2e–f. Comparing the XRD diffraction patterns for the non-annealed films, shown in Fig. 2c and e, it can be seen that the addition of ZnO-NPs completely changed the microstructure of the P3HTi films, introducing disturbances in the arrangement of both the polymer lamella system and the system of π stacked conjugated polymer units. This is clearly visible in Fig. 2e in the form of two broad contours in the range of 2θ angles of 5–9 and 12–16°, respectively. In turn, addition of PC70BM increases the degree of amorphousness of the films, as shown in Fig. 2g. However, this is not a surprise as the PC70BM70 powder itself is also amorphous. It should be noted that, similarly to the polymer films themselves, the non-annealed P3HT3:PC70BM film, i.e. the one with the lowest polymer molecular weight, shows by far the highest degree of amorphism. For this film, a clearly smaller order of the polymer lamellae can be observed. Heat treatment restores order in the films of the P3HTi:PC70BM mixture, as shown in Fig. 2h, and here again the P3HT3:PC70BM film shows the highest order.

Finally, the XRD patterns of P3HTi:PC70BM:ZnO-NPs are shown in Fig. 2i and j. It can be seen that the addition of ZnO-NPs to the P3HTi:PC70BM70 solutions causes a lack of order in the composite layers. At the same time, in each of these layers, three characteristic peaks of ZnO-NPs crystals are clearly visible, at 2θ from 28 to 38°. Moving on to conclusions important due to the main goal of our research. Note that the XRD diffraction patterns of the P3HT:PC70BM layers shown in Fig. 2 and their ZnO-NP composites shown in Fig. 2j, contain three peaks labeled (100), (200) and (300). However, unlike Fig. 2, they do not have a peak (010) indicating *π*-stacking. Thus, they point to a structural arrangement called an edge-on. Which is more favorable for organic field effect transistors than OSCs.

### Ellipsometry

**Figure 3:**
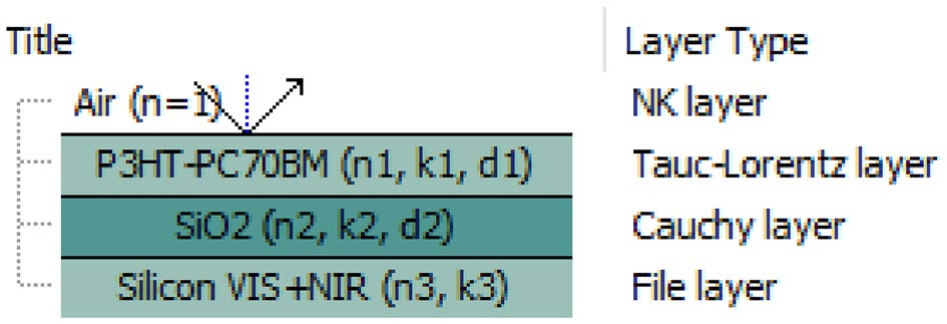
Optical model for the analysis of ellipsometric measurements.

Ellipsometry measures the complex reflectance ratio $\left( {\rho = e^{i\Delta } \tan \psi } \right)$, where *Ψ* and *Δ* are the ellipsometric angles. On the other hand, *ρ* depends on the complex dielectric functions of a particular optical system and must be determined theoretically. In our case, the optical system consists of four optical layers and is depicted in Fig. 3. Ellipsometric measurements were carried out on samples deposited on silicone substrates (coated with 400 nm silicon oxide) in VTSE and VASE modes. The first mode was used as a method of thermal analysis to determine thickness changes under the influence of temperature change and to determine the glass transition temperature of layers of mixtures and their composites with ZnO-NPs. The second method (VASE) was used to determine the optical properties of investigated films, at room temperature. Four Tauc-Lorentz oscillators were used to determine the optical properties of these films in the range of 250–2500 nm.

The single Tauc-Lorentz oscillator is expressed by the following formula:

1 $$\varepsilon_{2} \left( E \right) = \left\{ \begin{gathered} \frac{{AE_{0} C\left( {E - E_{g} } \right)^{2} }}{{\left( {E^{2} - E_{0}^{2} } \right) + C^{2} E^{2} }}\frac{1}{E};\quad E > E_{g} \hfill \\ 0;\quad \quad \quad \quad \quad \quad \quad \quad \quad \;\;E \le E_{g} \hfill \\ \end{gathered} \right.,$$

2 $$\varepsilon_{1} \left( E \right) = \varepsilon_{1} \left( \infty \right) + \frac{2}{\pi }P\mathop \int \limits_{{E_{g} }}^{\infty } \frac{{x\varepsilon_{2} \left( x \right)}}{{x^{2} - E^{2} }}dx,$$

where *ɛ*_1_ and *ɛ*_2_ are real and imaginary part of the dielectric constant and *E*_*0*_ is the peak transition energy, *C* is the broadening term, *E*_g_ is the energy gap and *P* stands for the Cauchy principal part of the integral. An additional fitting parameter, ${\varepsilon }_{1}\left(\infty \right)$, has been included in Eq. (2). The extinction coefficients *k*, refractive indices *n* and films thickness *d* were determined using Spectra Ray 3 software, note that

*ɛ*_1_ + *i ɛ*_2_ = (*n* + *ik*)^2^.

**Figure 4:**
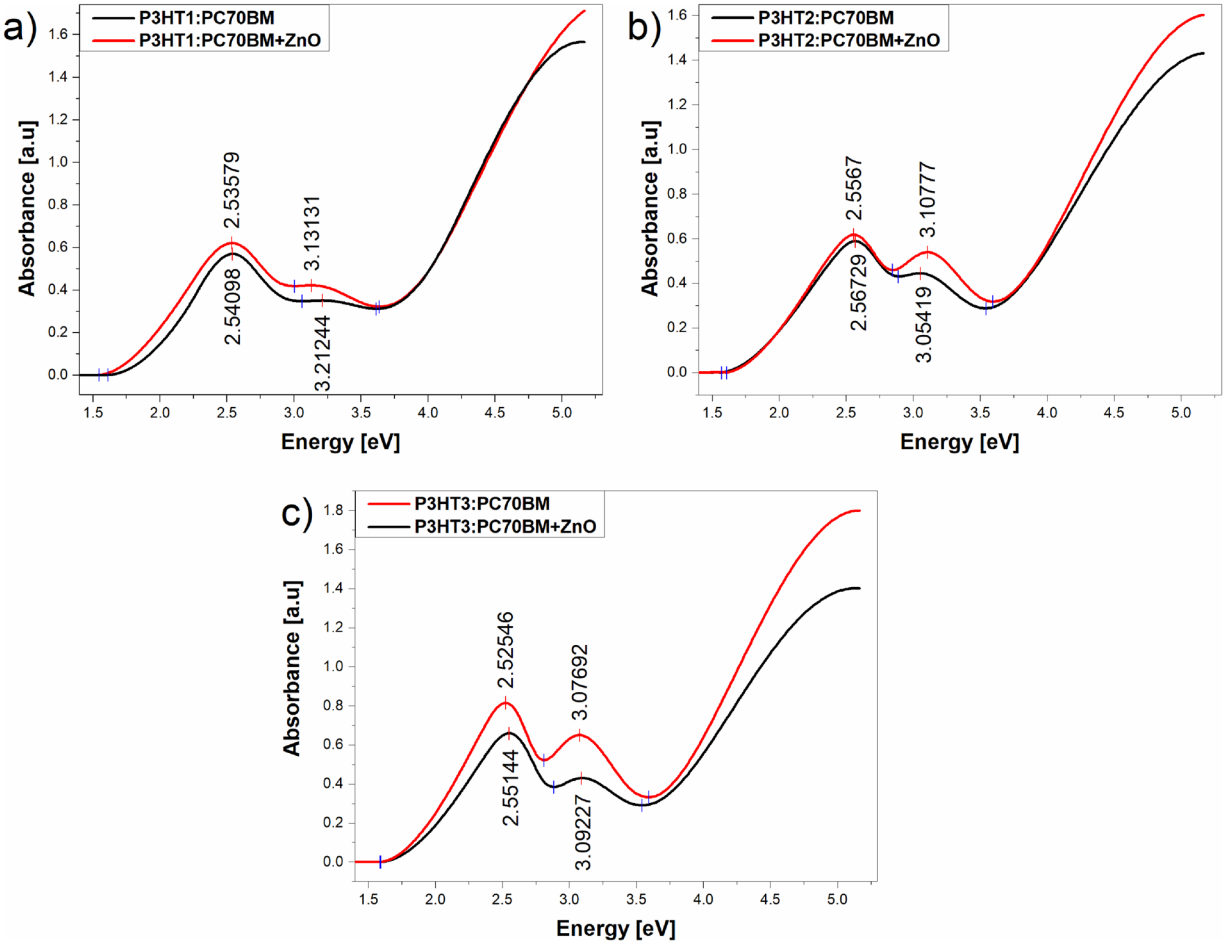
Absorbance for P3HT:PC70BM films and its composites with ZnO-NPs.

The absorption spectra of blends and composite films were determined on the basis of the ellipsometrically determined extinction coefficients as these quantities are related by the following simple linear relationship:

3 $$\alpha = \frac{4\pi k}{\lambda },$$

where $\alpha$ is the absorption coefficient. The spectra of the films of the P3HT:PC70BM blend were compared in Fig. 4 with those of the composites in the range of 1.4–5.5 eV. Two distinct absorption bands were observed in all cases. The first, low-energy absorption band, located at around 2.55 eV originates from electron transitions between π → π* molecular orbitals of P3HT, while the second band, with maximum at around 3.1 eV is coming from the electron transition in PC70BM, similarly like described in^32^. In all cases, the position of the first peak maximum does not change. The value of the second band maximum is slightly shifted in all composite spectra. The maximum values of these absorption bands are marked in Fig. 4a–c and it can be seen that the sharpening of these bands gradually increases with decreasing molecular weight of the P3HT used. The biggest difference can be seen in the case of P3HT3:PC70BM:ZNO-NPs composites. In addition, the intensity of the second absorption band increases with the decrease in the molar mass of P3HT in the case of composites.

This sequence also corresponds to the formation of P3HT lamellae crystallites and the overall increasing structural order in these films, as evidenced by the XRD studies reported above.

**Figure 5:**
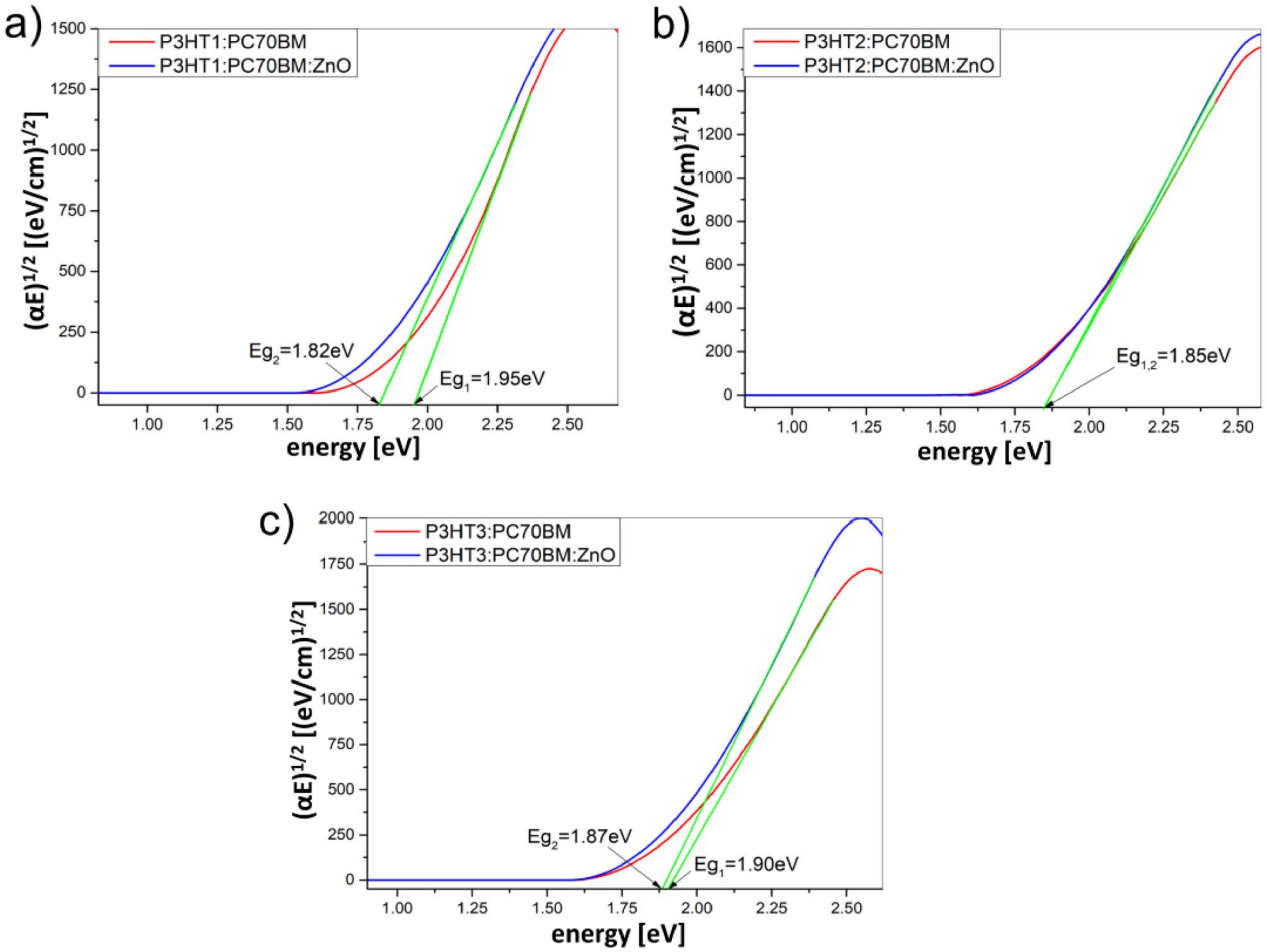
Determination of energy gaps (*E*_g_) for P3HT:PC70BM and its composites with ZnO-NPs thin films containing: (**a**) P3HT1, (**b**) P3HT2 and (**c**) P3H3.

Then, based on the obtained absorption spectra, we determined and compared the bandgap of P3HT:PC70BM mixtures and the corresponding composite layers, because the Tauc relationship^33^ can also be used to determine the energy bandgap of amorphous and nanocrystalline semiconductor materials^31,32^. The energy gaps were determined by the linear extrapolation to the plot (*αE*)^1/2^ versus photon energy *E*, what is presented in Fig. 5a–c. For all the tested composite layers, the presence of ZnO nanoparticles reduces the energy gap value compared to their counterparts in the P3HTi:PC70BM layers, see Table 2.

**Table 2:** Values of determined energy gaps *E*_*g*_.

	Blend *E*_g_ (eV)	ZnO-NPs composite *E*_g_ (eV)
P3HT1	1.95	1.82
P3HT2	1.85	1.85
P3HT3	1.90	1.87

**Figure 6:**
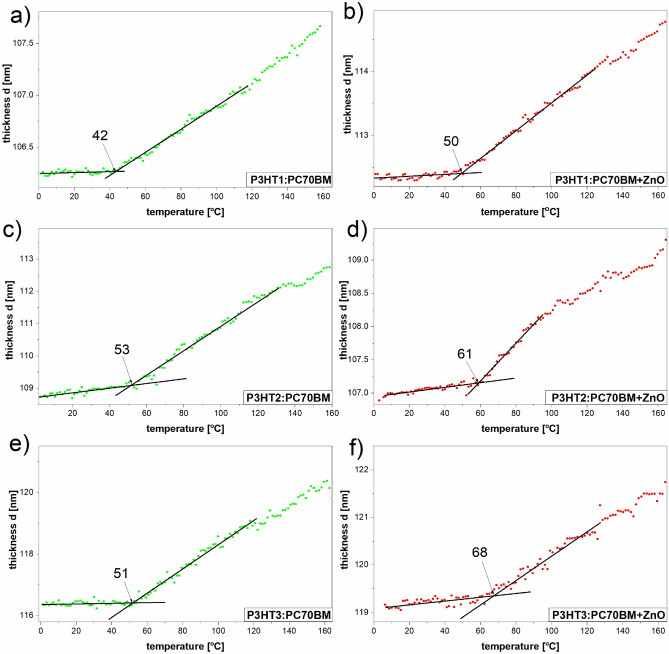
Film thickness as temperature function of P3HTi:PC70BM and their ZnO composites, where *i* = 1 (**a**, **b**), *i* = 2 (**c**, **d**) and *i* = 3 (**e**, **f**).

The thickness of the tested layers at different temperatures, *d*(*T*), was determined in their range of spectral transparency (800–930 nm) using the Cauchy model^9^ and Spectra Ray 3 software, as in our previous work^29,30^. The spectral dispersion of the optical parameters is described by the Cauchy model as follows:

4 $$n\left( {\lambda ,T} \right) = n_{0} \left( T \right) + C_{0} \frac{{n_{1} \left( T \right)}}{{\lambda^{2} }} + C_{1} \frac{{n_{2} \left( T \right)}}{{\lambda^{4} }},$$

5 $$k\left( {\lambda ,T} \right) = k_{0} \left( T \right) + C_{0} \frac{{k_{1} \left( T \right)}}{{\lambda^{2} }} + C_{1} \frac{{k_{2} \left( T \right)}}{{\lambda^{4} }},$$

where now temperature-dependent quantities *n*_i_ and *k*_*i*_, with *i* = 0, 1 and 2, are the model (fitting) parameters and the coefficients *C*_*0*_ and *C*_*1*_ are the numerical constants. The determined layer thicknesses as a function of temperature are presented in Fig. 6a–f.

**Figure 7:**
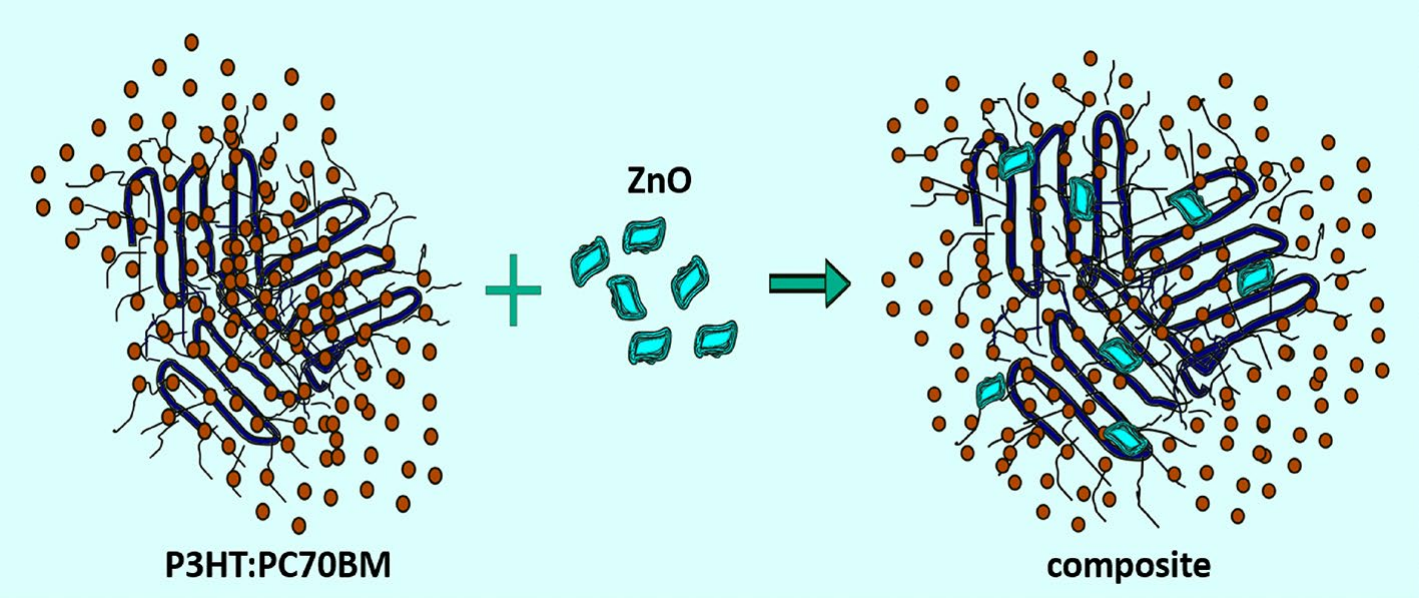
Scheme of P3HT:PC70BM:ZnO-NPs composite.

As can be seen, in relatively wide temperature ranges *d*(*T*) can be well described by linear functions. Moreover, thermal transitions are clearly visible, in which the value of the slope of these lines changes sharply. And this, in turn, indicates a sudden change in the value of the linear thermal expansion coefficient of the tested layers, i.e. a glass transition. Therefore, we consider the temperature at which these changes occur as glass transition temperature *T*_*g*_. In the case of the blend of PC70BM with P3HT1 of the highest molar mass (Mw = 65.2 kDa), the value of the determined glass transition temperature is around 42 °C. The glass transition temperature value of P3HT1:PC70BM:ZnO-NPs composite is around 50 °C. The *T*_*g*_ value determined for the P3HT2:PC70BM blend (where the molar mass of P3HT2 = 54.2 kDa) is at around 53 °C, and for the corresponding composite around 61 °C. For the PC70BM blend with P3HT3 i.e. with the lowest molar mass (Mw = 34.1 kDa), the value of the determined glass transition temperature is around 51 °C, and *T*_*g*_ of the corresponding blend approximately 68 °C. In this last case, there is the highest *T*_*g*_ difference between the blend and the corresponding composite. However, unambiguous determination of the influence of the P3HT molecular weight on the *T*_*g*_ of the tested films is difficult due to the relatively high uncertainty of the glass transition temperature determination, which is estimated at ± 5 °C. In the case of neat blends films, the values of *T*_*g*_ are better corresponding with regioregularities of P3HTi (*i* = 1–3), which are equal to 95.7, 94.2 and 94.7, respectively. However, we have observed the increase of average *T*_*g*_ value of composites (59.6 °C), compared to average *T*_*g*_ of blends (48.6 °C). This phenomena shows that added ZnO nanoparticles act as antiplasticizer. P3HT is a polymer with aliphatic side chains that can easily change positions with increasing temperature. Thus, their vitrification requires much lower temperatures. However, the glass transition temperature determined by us is much higher, because it is related to the mobility of the skeleton segments^34,35^. Therefore, we believe that the ZnO nanoparticles are embedded in the P3HT frameworks, thus stiffening the entire structure, as shown in Fig. 7. In other words, ZnO-NPs limit the repositioning of the main P3HT chains and contribute to an increase in the average value of the glass transition temperature.

### Surface imaging using scanning electron microscopy

**Figure 8:**
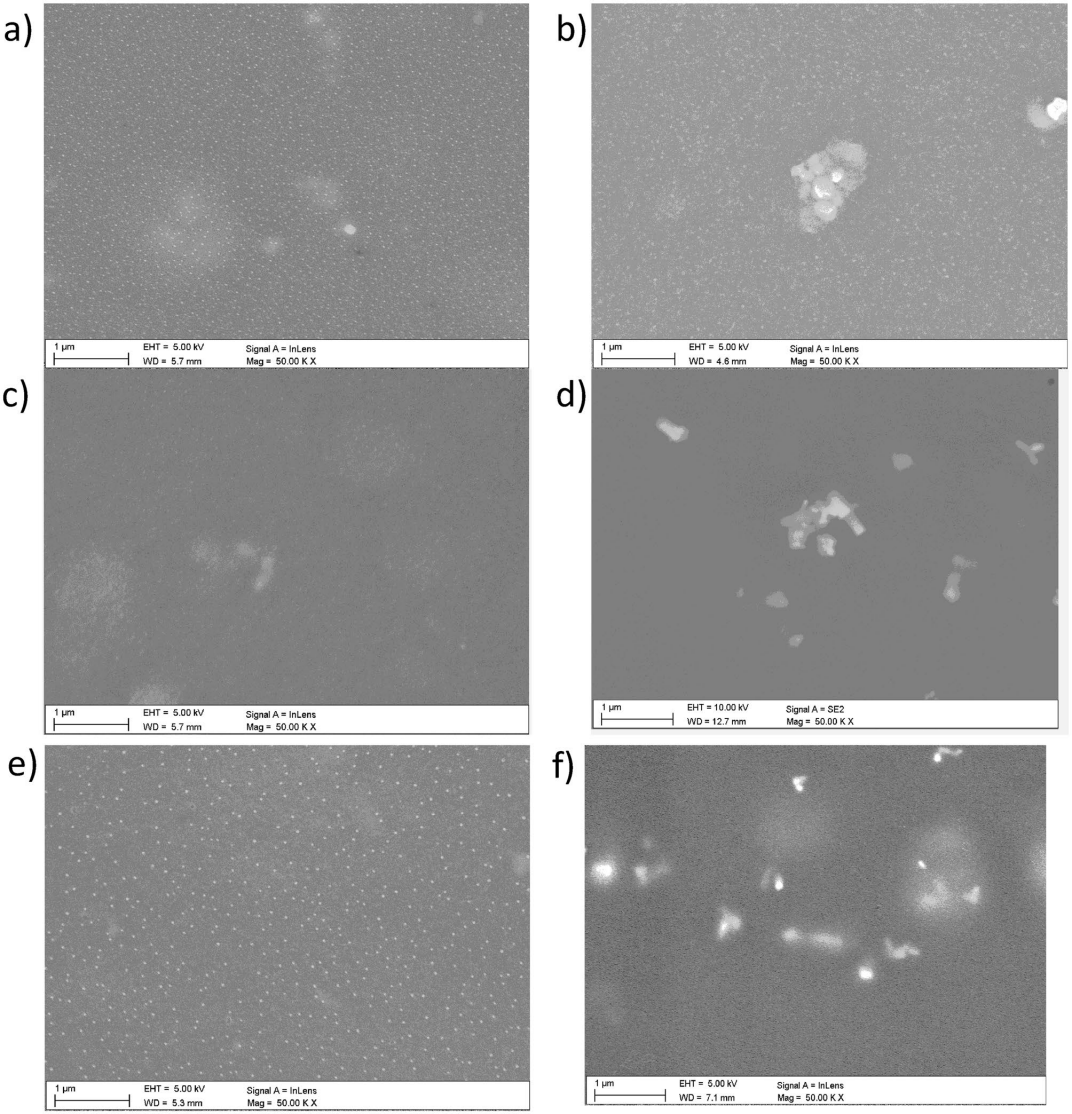
SEM pictures of: of P3HT1:PC70BM film (**a**) and its composite with ZnO (**b**); P3HT2:PC70BM blend (**c**) and its composite with ZnO (**d**); P3HT3:PC70BM (**e**) blend and its composite with ZnO (**f**).

The images of the film’s surface were taken for samples deposited on silicone substrates covered with a 400 nm SiO2 film. SEM surface imaging was performed on samples subjected to temperature tests, i.e. after heating and cooling them down. The surface images of the P3HTi:PC70BM blend films are shown in Fig. 8a, c and e. Dots from PC70BM can be seen on the surface of all samples.

The surface of these films is fairly homogeneous with uniformly distributed PC70BM clusters and less contrasted P3HT:PC70BM clusters. In clear contrast, the surface of composites, Fig. 8b, d and f shows agglomerates of ZnO-NPs that were formed despite the homogenization of prepared solutions from which the films were deposited. The formation of nanoparticle agglomerates is due to their natural tendency to minimize surface energy while increasing the diameter of the cluster. This effect is caused by the competition of two forces—the Van der Waals binding force and the repulsive electrostatic interaction. The agglomeration effect is exacerbated when the nanoparticles are in a solution of relatively low viscosity and the Van der Waals interaction prevails^36^.

### Organic photovoltaic (OPV) devices

The same batches of P3HT:PC70BM mixtures and their composite solutions with ZnO were used to prepare active films in bulk-heterojunction (BHJ) photovoltaic cells. The thickness of the active films was in the range of 100–150 nm, and the OPV structures had the following arrangement:

**ITO/PEDOT: PSS/P3HT: PC70BM/Alu** and **ITO/PEDOT: PSS/P3HT: PC70BM: ZnO-NPs/Alu**

The average values of the parameters, related to the efficiency of the obtained cells are presented in Table 3. They are: open circuit voltage (*V*_*oc*_), short circuit current (*I*_*sc*_), fill-factor (*FF*), and *PCE*. Comparing *PCE* for the P3HTi:PC70BM mixtures, it can be seen that they increased with the decrease in the molecular weight of P3HTi from 2.2 to 2.9%. However, in the case of composite films, we observe the opposite behavior. The efficiency values of cells with active layers containing ZnO-NPs were lower and ranged from 1.7 to 1.1%. On the other hand, the *V*_*oc*_ of all prepared OSC structures ranged from 0.51 to 0.43 V, with as many as 5 of them having *V*_*oc*_ not lower than 0.48 V. Taking into account the accuracy of the measurement, it can be assumed that regardless of the polymer molecular weight and the presence of ZnO-NPs, this value did not change. This is consistent with the fact that *V*_*oc*_ of P3HT:PCBM-based BHJ OSCs is proportional to the difference in the molecular energy levels of HOMO donors (${E}_{HOMO}^{donor}$) and LUMO acceptors (${E}_{LUMO}^{aceptor}$) and some empirical correction $\Delta V$ determining *V*_*oc*_ drop^37^. The open circuit voltage can then be expressed as:

6 $$V_{oc} = \frac{1}{e}\left( {E_{HOMO}^{donor} - E_{LUMO}^{aceptor} } \right) - \Delta V$$

where $e$ denote elementary charge. Similarly, the fill factor has a constant value of 0.44 for almost all cells. Only the OSC from the P3HT1:PC70BM blend has a clearly lower value of *FF* = 0.33. It should also be noted that Table 3 clearly shows a decrease in *I*_*sc*_ value in the solar cells containing ZnO-NPs. Given that fill factor is defined as follows:

7 $$FF = \frac{{V_{m} *I_{m} }}{{V_{oc} *I_{sc} }},$$

where *V*_*m*_ and *I*_*m*_ are voltage and current at maximum power. This means that with a constant value of *FF* and a variable *I*_*sc*_ at the same time, the maximum power of a given OSC must change accordingly. This is reflected in the power conversion efficiency which can be expressed by fill factor and the product $V_{oc} *I_{sc}$ as follows:

8 $$PCE = FF*\frac{{V_{oc} *I_{sc} }}{{P_{in} }},$$

where ${P}_{in}$ is incident solar power. We attribute the reduction of the short-circuit current I_sc_ to the presence of electron traps associated with oxygen defects (vacancies) in ZnO-NPs, which may lead to a decrease in the efficiency of the prepared photovoltaic cells^18^.

**Table 3:** Average performance of prepared solar cells films depending on P3HT molecular weight and on ZnO-NPs content.

Active film	V_OC_ (V)	I_SC_ (mA/cm^2^)	FF (1)	η (%)
P3HT1/PC70BM	0.51 ± 0.05	12.72 ± 0.50	0.33 ± 0.02	2.19 ± 0.20
P3HT1/PC70BM + 10% of ZnO	0.48 ± 0.05	7.86 ± 0.50	0.45 ± 0.02	1.74 ± 0.20
P3HT2/PC70BM	0.49 ± 0.05	10.74 ± 0.50	0.44 ± 0.02	2.37 ± 0.20
P3HT2/PC70BM + 10% of ZnO	0.48 ± 0.05	4.87 ± 0.50	0.43 ± 0.02	1.06 ± 0.20
P3HT3/PC70BM	0.49 ± 0.05	13.44 ± 0.50	0.42 ± 0.02	2.89 ± 0.20
P3HT3/PC70BM + 10% of ZnO	0.43 ± 0.05	5.23 ± 0.50	0.44 ± 0.02	1.05 ± 0.20

**Figure 9:**
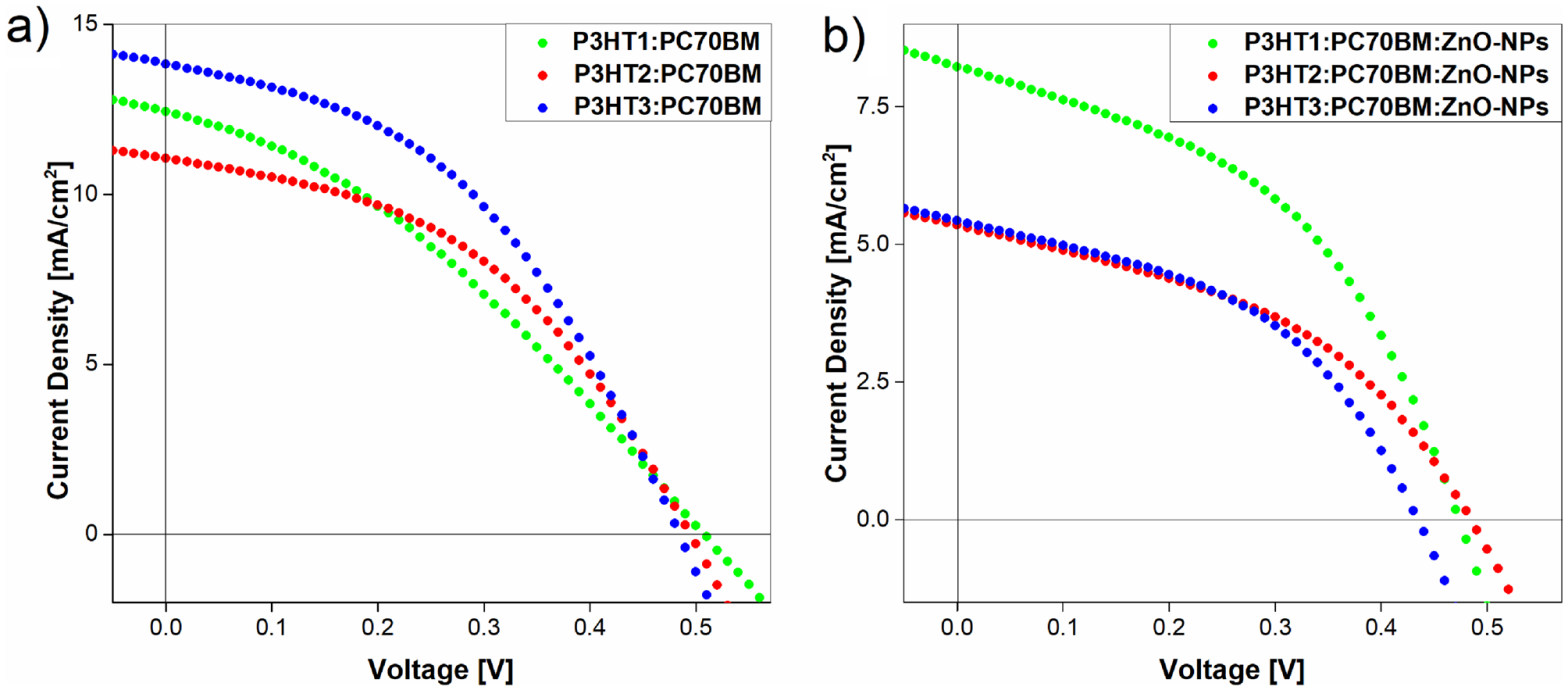
J-V characteristic curves of P3HTi:PC70BM (**a**) and P3HTi:PC70BM:ZnO-NPs (**b**) devices, where *i* = 1–3.

For completeness, we also present exemplary characteristics of organic solar cells with active layers P3HTi:PC70BM and P3HTi:PC70BM:ZnO-NPs (*i* = 1–3), respectively, as shown in Fig. 9a–b shown in right panel (b).

Generally, the efficiency values of cells with P3HTi:PC70BM mixtures are not high, undoubtedly an important reason for this is the ordering of the P3HT lamellae in the edge-on arrangement. Nevertheless, the conducted research shows a systematic trend. In particular, we note that the highest performance was obtained for the P3HT3:PC70BM mixture, which gave the most amorphous film before annealing.

## Conclusions

In this work, films made of P3HTi of various molecular weights, PC70BM, their mixtures and composites with ZnO-NPs were investigated. The samples were characterized by X-ray diffraction, variable-angle spectroscopic ellipsometry, temperature-dependent ellipsometry and scanning electron microscopy.

The addition of ZnO-NPs powder affected the thermal properties and morphology of the tested layers, which was confirmed by various experimental techniques. Based on the results of X-ray tests, it can be concluded that the internal order of the obtained layers was influenced mainly by two factors—the molecular weight of the polymer used in the P3HTi:PC70BM mixtures and the addition of ZnO nanoparticles.

The highest ordering of the internal microstructure occurred in the case of blend films prepared with the use of P3HT3, with a lower molecular weight. The introduction of ZnO nanoparticles to the P3HT polymer disrupted the structural order of the layer. Annealing restored structural order. In the case of layers of P3HTi:PC70BM:ZnO-NPs composites, the effect is very similar, as evidenced by the value of their glass transition temperatures, which are higher than in the case of layers of corresponding P3HT:PC70BM mixtures without ZnO-NPs. The average value of the glass transition temperature of the composite layers was approx. 60 °C, while the average *T*_*g*_ of the layers of the mixtures without NPs was approx. 45 °C. The optical and thermal ellipsometric measurements showed that composite films had slightly better physical properties compared to the blends. It was observed that the layers of P3HT:PC70BM:ZnO-NPs had higher absorption intensity than the layers of pure blends.

This is quite important due to the amount of light absorbed by photovoltaic cells. The determined value of the optical bandgap of the composite layers showed a decrease, and thus an improvement in the conjugation length and conductivity, in relation to their counterparts without NPs can be expected. In the case of solar cells, where just blends were used as active layers, the power efficiency was increasing with the decreasing molecular weight of P3HT, which can be associated with a higher degree of internal order. In turn, the efficiency of solar cells, in which the active film was a composite with ZnO-NPs was lower than the efficiency of corresponding cells prepared from pure blend films. We attributed this reduction in *PCE* to the presence of electron traps associated with oxygen defects (vacancies) in ZnO-NPs.

## Data Availability

The datasets used and/or analyzed during the current study available from the corresponding author on reasonable request.

## References

[1] Giannouli M (2021). Current status of emerging PV technologies: A comparative study of dye-sensitized, organic, and perovskite solar cells. Int. J. Photoenergy.

[2] Gong J, Sumathy K, Qiao Q, Zhou Z (2017). Review on dye-sensitized solar cells (DSSCs): Advanced techniques and research trends. Renew. Sustain. Energy Rev..

[3] Giannouli M, Drakonakis VM, Savva A, Eleftheriou P, Florides G, Choulis SA (2015). Methods for improving the lifetime performance of organic photovoltaics with low-costing encapsulation. Chem. Phys. Chem..

[4] Cui Y, Yao H, Hong L (2020). Organic photovoltaic cell with 17% efficiency and superior processability. Nat. Sci. Rev..

[5] Chen LX (2019). Organic solar cells: Recent progress and challenges. ACS Energy Lett..

[6] Abdulrazzaq OA, Saini V, Bourdo S, Dervishi E, Biris AS (2013). Organic solar cells: A review of materials, limitations, and possibilities for improvement. Part. Sci. Technol..

[7] Bednarski H, Hajduk B, Domański M (2018). Unveiling of polymer/fullerene blend films morphology by ellipsometrically determined optical order within polymer and fullerene phases. J. Polym. Sci. Part B Polym. Phys..

[8] Hajduk B, Bednarski H, Jarząbek B, Nitschke P, Janeczek H (2020). Phase diagram of P3HT:PC70BM thin films based on variable-temperature spectroscopic ellipsometry. Polym. Test..

[9] Hajduk B, Bednarski H, Trzebicka B (2020). Temperature-dependent spectroscopic ellipsometry of thin polymer films. J. Phys. Chem. B.

[10] Pearson AJ, Wang T, Jones RAL (2012). Rationalizing phase transitions with thermal annealing temperatures for P3HT:PCBM organic photovoltaic devices. Macromolecules.

[11] Yu J, Zheng Y, Huang Y (2014). Towards high performance organic photovoltaic cells: A review of recent development in organic photovoltaics. Polymers.

[12] Park S, Seo Y-S, Shin WS, Moon S-J, Hwang J (2016). Rapid and checkable electrical post-treatment method for organic photovoltaic devices. Sci. Rep..

[13] Weng K, Ye L, Zhu L (2020). Optimized active film morphology toward efficient and polymer batch insensitive organic solar cells. Nat. Commun..

[14] Cui C, Li Y (2021). Morphology optimization of photoactive films in organic solar cells. Aggregate.

[15] Swart HC, Ntwaeaborwa OM, Mbule PS (2015). P3HT: PCBM based solar cells: A short review focusing on ZnO nanoparticles buffer layer, post-fabrication annealing and an inverted geometry. J. Mater. Sci. Eng. B.

[16] Liu C, Zhang D, Li Z (2017). Decreased charge transport barrier and recombination of organic solar cells by constructing interfacial nanojunction with annealing-free ZnO and Al films. ACS Appl. Mater. Interfaces.

[17] Wanninayake AP, Church BC, Abu-Zahra N (2016). Effect of ZnO nanoparticles on the power conversion efficiency of organic photovoltaic devices synthesized with CuO nanoparticles. AIMS Mater. Sci..

[18] Kim O, Kwon J, Kim S (2019). Effect of PVP-capped ZnO nanoparticles with enhanced charge transport on the performance of P3HT/PCBM polymer solar cells. Polymers.

[19] Mohtaram F, Borhani S, Ahmadpour M (2020). Electrospun ZnO nanofiber interlayers for enhanced performance of organic photovoltaic devices. Sol. Energy.

[20] Ikram M, Murray R, Imran M, Ali S, Ismat Shah S (2016). Enhanced performance of P3HT/(PCBM:ZnO:TiO_2_) blend based hybrid organic solar cells. Mater. Res. Bull..

[21] Chonsut T, Pratontep S, Keawprajak A, Kumnorkaew P, Kayunkid N (2016). Improvement of efficiency of polymer-zinc oxide hybrid solar cells prepared by rapid convective deposition. Appl. Mech. Mater..

[22] Bi S, Li Y, Liu Y, Ouyang Z, Jiang C (2018). Physical properties of 2D and 3D ZnO materials fabricated by multi-methods and their photoelectric effect on organic solar cells. J. Sci. Adv. Mater. Devices.

[23] Ciammaruchi L, Oliveira R, Charas A (2018). Stability of organic solar cells with PCDTBT donor polymer: An interlaboratory study. J. Mater. Res..

[24] Rosmani CH, Zainurul AZ, Rusop M, Abdullah S (2017). The optical and electrical properties of polymer poly (3-Hexylthiophene) P3HT by heat treatment. Adv. Mater. Res..

[25] Holliday S, Ashraf RS, Wadsworth A (2016). High-efficiency and air-stable P3HT-based polymer solar cells with a new non-fullerene acceptor. Nat. Commun..

[26] Li Y, Qi D, Song P, Ma F (2015). Fullerene-based photoactive films for heterojunction solar cells: Structure, absorption spectra and charge transfer process. Materials.

[27] Jaglarz J, Małek A, Sanetra J (2018). Thermal dependence of optical parameters of thin polythiophene films blended with PCBM. Polymers.

[28] Hajduk B, Jarka P, Tański T (2022). an investigation of the thermal transitions and physical properties of semiconducting PDPP4T:PDBPyBT blend films. Materials.

[29] Hajduk B, Bednarski H, Jarząbek B, Janeczek H, Nitschke P (2018). P3HT:PCBM blend films phase diagram on the base of variable-temperature spectroscopic ellipsometry. Beil. J. Nanotechnol..

[30] Hajduk B, Bednarski H, Domański M, Jarząbek B, Trzebicka B (2020). Thermal transitions in P3HT:PC60BM films based on electrical resistance measurements. Polymers.

[31] Jarząbek B, Nitschke P, Godzierz M, Palewicz M, Piasecki T, Gotszalk TP (2022). Thermo-optical and structural studies of iodine-doped polymer: Fullerene blend films, used in photovoltaic structures. Polymers.

[32] Jarząbek B, Nitschke P, Hajduk B, Domański M, Bednarski H (2020). In situ thermo-optical studies of polymer: Fullerene blend films. Polym. Test..

[33] Tauc J, Menth A (1972). States in the gap. J. Non Cryst. Solids.

[34] Xie R, Weisen AR, Lee Y (2020). Glass transition temperature from the chemical structure of conjugated polymers. Nat. Commun..

[35] Xie R, Lee Y (2017). Glass transition temperature of conjugated polymers by oscillatory shear rheometry. Macromolecules.

[36] Tsuda A, Konduru NV (2016). The role of natural processes and surface energy of inhaled engineered nanoparticles on aggregation and corona formation. NanoImpact.

[37] Scharber MC, Sariciftci NS (2013). Efficiency of bulk-heterojunction organic solar cells. Prog. Polym. Sci..

